# Surfactant protein A mediates pulmonary clearance of *Staphylococcus aureus*

**DOI:** 10.1186/s12931-014-0085-2

**Published:** 2014-08-05

**Authors:** Nils T Veith, Thomas Tschernig, Birgitt Gutbier, Martin Witzenrath, Carola Meier, Michael Menger, Markus Bischoff

**Affiliations:** 1 Institute of Anatomy and Cell Biology, Kirrberger Straße, Homburg/Saar, 66421, Germany; 2 Department for Infectious and Pulmonary Disease, Charité Universitätsmedizin Berlin, Berlin, 10117, Germany; 3 Institute for Clinical and Experimental Surgery, University of Saarland, Homburg/Saar, Germany; 4 Institute for Microbiology and Hygiene, Saarland University, Medical Faculty, Homburg/Saar, 66421, Germany

## Abstract

Surfactant protein A has been shown to enhance opsonization and clearance of *Staphylococcus aureus in vitro*. Here, the phagocytosis of alveolar *S. aureus* was investigated *in vivo* using intravital microscopy. Fluorescence labelled *S. aureus* Newman cells were intratracheally administered to anesthetized mice and the alveolar surface was observed for fifteen minutes. Confirming previously reported *in vitro* data, surfactant protein A-deficient mice showed a significantly reduced uptake of bacteria compared to wild-type mice.

## Findings

**Figure 1:**
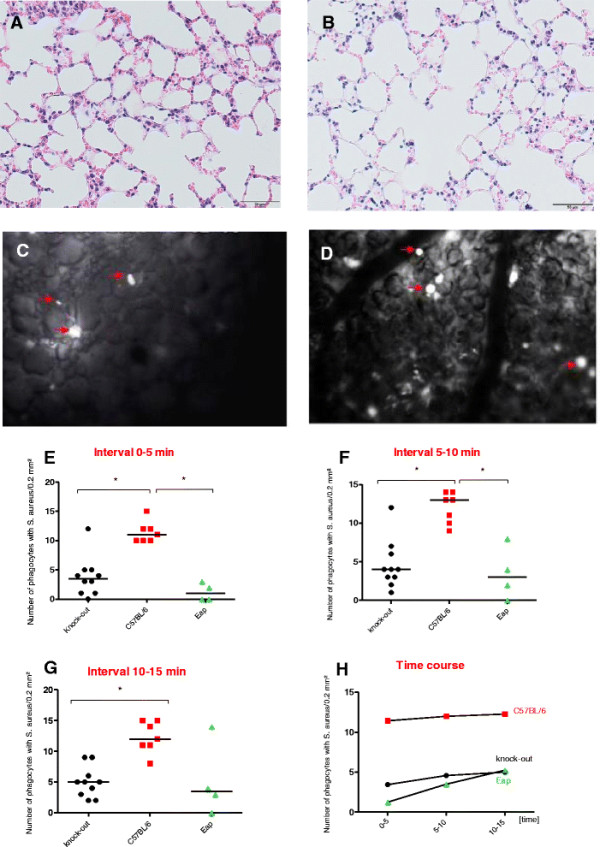
**Ingestion of fluorescence-labelled*S. aureus*Newman and Newman*eap*cells by alveolar macrophages over time in wild-type and SP-A**^**−/−**^**mice (n = 4–10).(A, B)** Normal lung histology was observed after the experiment in the SP-A deficient animals **(A)** and in the wild-type animals **(B)**. **(C, D)** A representative image of intravital microscopy shows bright, shining phagocytes in the alveoli which have ingested fluorescent bacteria. Clearly less of those phagocytes could be seen in SP-A deficient animals **(C)** compared to wild-type animals **(D)**. **(E-H)** The count of such cells is presented in three subsequent intervals **(E, F, G)**. A slight increase was observed over time **(H)**.

Collectins are a part of surfactant proteins and have significant functions in the opsonization and clearance of bacteria in the pulmonary microenvironment. Absence of surfactant protein A (SP-A) leads to increased susceptibility for bacterial infections [1]–[3]. Interestingly, SP-A binds to the *S. aureus* extracellular adherence protein, Eap, thereby enhancing phagocytosis and killing of *S. aureus* by alveolar macrophages [4]. In this study the role of SP-A in the phagocytosis of *S. aureus* in the peripheral alveoli was investigated *in vivo* by intravital microscopy. Fluorescence labelling and intratracheal inoculation of *S. aureus* as well as intravital microscopy were performed essentially as described before [5]. Briefly, exponential growth phase cells of *S. aureus* strain Newman were labelled using 5-[6]-carboxyfluorescein diacetate succinimidyl ester. The suspension (100 μl containing 2 × 10^8^ colony forming units) was injected into the inspiration limb. Mice (7 wild-type and 10 SP-A^−/−^ on C57BL/6 background, 20 to 25 g body weight; [1]) were anesthetized and ventilated after tracheotomy. Fluorescence-labelled viable bacteria were administered and a thoracotomy was performed. Imaging commenced thirty minutes after the application of bacteria. Three 5 min intervals were recorded and then the experiment was concluded. The complete anterior part of the right thorax was removed surgically. A micromanipulator was used to position a cover-glass horizontally over the surface of the lung. By using a drop of warm saline the lung surface was attached by adhesion forces to the lower surface of the cover-glass. Ventilation was discontinued occasionally for up to 15 seconds to avoid ventilation-induced movements and to obtain a comparable inflation of the lung (PEEP 4 cm H_2_O). The upper right lung lobe was imaged by means of a fluorescence microscope and a filter set for blue light (450–490 nm) epi-illumination. Microscopic images were recorded by means of a charge-coupled device video camera and digitally recorded for subsequent off-line analyses. For each time interval (0–5 min, 5–10 min, 10–15 min) randomly selected fields (3 per interval and animal) were chosen to determine the number of active cells of phagocytosis. Data are given as mean values ± standard deviation (SD). At the end of the experiment all lungs revealed normal morphology. No edema was found after this short viewing period (Figure 1A-B). The fluorescent staphylococci reached the alveoli and were ingested within minutes. Phagocytes which had internalized labelled bacteria shone brightly in the outer alveoli (Figure 1C-D). As known from previous studies, most of the phagocytes detected early after inoculation are alveolar macrophages [5]. In all intervals a significantly lower number of phagocytes filled with staphylococci were found in SP-A deficient mice than in WT (Figure 1E-F), confirming recent findings indicating SP-A to mediate pulmonary clearance of *Staphylococcus aureus*[4]. To proove that interaction of SP-A with the bacterial cell wall-associated Eap contributes to phagocytosis, four experiments were performed using C57BL/6 mice and cells of the Eap-deficient *S. aureus* Newman derivative mAH12 (Newman *eap::ermB*; [6]) under identical conditions. Here, results similar to those with the SP-A deficient mice and *S. aureus* Newman wild type cells were obtained (Figure 1E-H). These findings further support the above-mentioned study on the roles of SP-A and Eap in promoting phagocytosis of staphylococci by alveolar macrophages [4].

## Competing interests

The authors declare that they have no competing interests.

## Authors’ contributions

NTV, TT, BG and MW planned the experiments. NTV, TT and MB conducted the experiments. MB did the preparation of the bacteria. CM and MM discussed the data and partly wrote the manuscript. All authors read and approved the final manuscript.
